# Can Animal Models of Disease Reliably Inform Human Studies?

**DOI:** 10.1371/journal.pmed.1000245

**Published:** 2010-03-30

**Authors:** H. Bart van der Worp, David W. Howells, Emily S. Sena, Michelle J. Porritt, Sarah Rewell, Victoria O'Collins, Malcolm R. Macleod

**Affiliations:** 1 Department of Neurology, Rudolf Magnus Institute of Neuroscience, University Medical Centre Utrecht, Utrecht, The Netherlands; 2 National Stroke Research Institute & University of Melbourne Department of Medicine, Austin Health, Melbourne, Australia; 3 Department of Clinical Neurosciences, University of Edinburgh, Edinburgh, United Kingdom

## Abstract

H. Bart van der Worp and colleagues discuss the controversies and possibilities of translating the results of animal experiments into human clinical trials.

**Linked Research Article**

This Research in Translation discusses the following new study published in *PLoS Biology*:

Sena ES, van der Worp HB, Bath PMW, Howells DW, Macleod MR (2010) Publication bias in reports of animal stroke studies leads to major overstatement of efficacy. PLoS Biol 8(3): e1000344. doi:10.1371/journal. pbio.1000344

Publication bias confounds attempts to use systematic reviews to assess the efficacy of various interventions tested in experiments modeling acute ischemic stroke, leading to a 30% overstatement of efficacy of interventions tested in animals.

**Summary Points**

- The value of animal experiments for predicting the effectiveness of treatment strategies in clinical trials has remained controversial, mainly because of a recurrent failure of interventions apparently promising in animal models to translate to the clinic.
- Translational failure may be explained in part by methodological flaws in animal studies, leading to systematic bias and thereby to inadequate data and incorrect conclusions about efficacy.
- Failures also result because of critical disparities, usually disease specific, between the animal models and the clinical trials testing the treatment strategy.
- Systematic review and meta-analysis of animal studies may aid in the selection of the most promising treatment strategies for clinical trials.
- Publication bias may account for one-third or more of the efficacy reported in systematic reviews of animal stroke studies, and probably also plays a substantial role in the experimental literature for other diseases.
- We provide recommendations for the reporting of aspects of study quality in publications of comparisons of treatment strategies in animal models of disease.

Animal experiments have contributed much to our understanding of mechanisms of disease, but their value in predicting the effectiveness of treatment strategies in clinical trials has remained controversial [1]–[3]. In fact, clinical trials are essential because animal studies do not predict with sufficient certainty what will happen in humans. In a review of animal studies published in seven leading scientific journals of high impact, about one-third of the studies translated at the level of human randomised trials, and one-tenth of the interventions, were subsequently approved for use in patients [1]. However, these were studies of high impact (median citation count, 889), and less frequently cited animal research probably has a lower likelihood of translation to the clinic. Depending on one's perspective, this attrition rate of 90% may be viewed as either a failure or as a success, but it serves to illustrate the magnitude of the difficulties in translation that beset even findings of high impact.

Recent examples of therapies that failed in large randomised clinical trials despite substantial reported benefit in a range of animal studies include enteral probiotics for the prevention of infectious complications of acute pancreatitis, NXY-059 for acute ischemic stroke, and a range of strategies to reduce lethal reperfusion injury in patients with acute myocardial infarction [4]–[7]. In animal models of acute ischemic stroke, about 500 “neuroprotective” treatment strategies have been reported to improve outcome, but only aspirin and very early intravenous thrombolysis with alteplase (recombinant tissue-plasminogen activator) have proved effective in patients, despite numerous clinical trials of other treatment strategies [8],[9].

## Causes of Failed Translation

The disparity between the results of animal models and clinical trials may in part be explained by shortcomings of the clinical trials. For instance, these may have had insufficient statistical power to detect a true benefit of the treatment under study. For practical or commercial purposes, the designs of some clinical trials have also failed to acknowledge the limitations of efficacy observed in animal studies, for example by allowing therapy at later time points when the window of opportunity has passed [10],[11]. Secondly, the failure of apparently promising interventions to translate to the clinic may also be caused by inadequate animal data and overoptimistic conclusions about efficacy drawn from methodologically flawed animal studies. A third possible explanation is the lack of external validity, or generalisability, of some animal models; in other words, that these do not sufficiently reflect disease in humans. Finally, neutral or negative animal studies may be more likely to remain unpublished than neutral clinical trials, giving the impression that the first are more often positive than the second. This article aims to address the possible sources of bias that threaten the internal and external validity of animal studies, to provide solutions to improve the reliability of such studies, and thereby to improve their translation to the clinic.

## Internal Validity

Adequate internal validity of an animal experiment implies that the differences observed between groups of animals allocated to different interventions may, apart from random error, be attributed to the treatment under investigation [12]. The internal validity may be reduced by four types of bias through which systematic differences between treatment groups are introduced (Table 1). Just like any clinical trial, each formal animal study testing the effectiveness of an intervention should be based on a well-designed study protocol addressing the design and conduct of the study, as well as the analysis and reporting of its results. Aspects of the design, conduct, and analysis of an animal experiment that help to reduce bias and to improve the reliability and reproducibility of the results are discussed below. As the impact of study quality has been studied much more extensively in clinical trials than in animal studies, the backgrounds and recommendations regarding these issues are largely based on the clinical CONsolidated Standards of Reporting Trials (CONSORT) statement, and to a smaller extent on published recommendations and guidelines for the conduct and reporting of animal studies of acute ischemic stroke [13]–[17].

**Table 1:** Four types of bias threatening internal validity.

Type of Bias	Definition	Solution
***Selection bias***	Biased allocation to treatment groups	Randomisation; allocation concealment
***Performance bias***	Systematic differences in care between the treatment groups, apart from the intervention under study	Blinding
***Detection (ascertainment, assessment, or observer) bias***	Systematic distortion of the results of a study that occurs when the person assessing outcome has knowledge of treatment assignment.	Blinding
***Attrition bias***	Unequal occurrence and handling of deviations from protocol and loss to follow-up between treatment groups	Blinding; intention-to-treat analysis

Adapted from [12],[13].

### Randomisation

To prevent selection bias, treatment allocation should be based on randomisation (Box 1), a method that is almost ubiquitous in clinical treatment trials. In part, this prevents the investigator from having to choose which treatment a particular animal will receive, a process which might result (consciously or subconsciously) in animals which are thought to do particularly well or particularly badly being overrepresented in a particular treatment group. Foreknowledge of treatment group assignment may also lead to selective exclusion of animals based on prognostic factors [13]. These problems can arise with any method in which group allocation is known in advance or can be predicted. Such methods include both the use of predetermined rules (e.g., assignment in alternation or on the basis of the days of the week) or of open randomisation schedules. Picking animals “at random” from their cages also has the risk of conscious or subconscious manipulation, and does not represent true randomisation.

**Box 1. Glossary**

- **Allocation concealment:** Concealing the allocation sequence from those assigning animals to intervention groups, until the moment of assignment.
- **Bias:** Systematic distortion of the estimated intervention effect away from the “truth,” caused by inadequacies in the design, conduct, or analysis of an experiment.
- **Blinding (masking):** Keeping the persons who perform the experiment, collect data, and assess outcome unaware of the treatment allocation.
- **Eligibility criteria:** Inclusion and exclusion criteria: the characteristics that define which animals are eligible to be enrolled in a study.
- **External validity:** The extent to which the results of an animal experiment provide a correct basis for generalisations to the human condition.
- **Intention-to-treat analysis:** Analysis of data of all animals included in the group to which they were assigned, regardless of whether they completed the intervention.
- **Internal validity:** The extent to which the design and conduct of the trial eliminate the possibility of bias.
- **Power:** The probability that a study will detect a statistically significant effect of a specified size.
- **Randomisation:** Randomly allocating the intervention under study across the comparison groups, to ensure that group assignment cannot be predicted.
- **Sample size:** The number of animals in the study

Definitions adapted from [13] and from Wikipedia (http://www.wikipedia.org, accessed on 9 November 2009).

Randomisation may appear redundant if the animals form a homogeneous group from a genetic and environmental perspective, as often is the case with rats and other rodents. However, it is not only the animal itself but mainly the induction of the disease that may give rise to variation. For example, there is a large variation in infarct size in most rat models of ischaemic stroke not only because of interindividual differences in collateral circulation—even in inbred strains—but also because in some animals the artery is occluded better than in others and because the models are inherently vulnerable to complications that may affect outcome, such as periprocedural hypotension or hypoxemia. It is because of this variation that randomisation, ideally occurring after the injury or disease has been induced, is essential.

In clinical trials, automated randomisation techniques such as random number generation are most commonly used, but manual methods (such as tossing a coin or throwing dice) are also acceptable as long as these cannot be manipulated. By preference, such manual techniques should be performed by an independent person.

### Blinding

In studies that are blinded throughout their course, the investigators and other persons involved will not be influenced by knowledge of the treatment assignment, thereby preventing performance, detection, and attrition bias. Knowledge of treatment assignment may subconsciously or otherwise affect the supply of additional care, outcome assessment, and decisions to withdraw animals from the experiment.

In contrast to allocation concealment (Box 1), blinding may not always be possible in all stages of an experiment, for example when the treatment under investigation concerns a surgical procedure. However, blinding of outcome assessment is almost always possible.

In clinical trials, the most common form of blinding is double blinding, in which the patients, the investigators, and the caregivers are unaware of the intervention assignment. Because the patient does not know which treatment is being administered, the placebo effect will be similar across the comparison groups. As animals are not susceptible to the placebo effect, double blinding is not an issue in animal studies. Notwithstanding the influence that unblinded animal handling can have on performance in neurobehavioural tasks [18], the fact that in some articles of animal studies “double blinding” is reported raises questions about the authors' knowledge of blinding as well as about the review and editorial processes of the journals in which the studies were published [19],[20].

### Sample Size Calculation

Selection of target sample size is a critical factor in the design of any comparison study. The study should be large enough to have a high probability of detecting a treatment effect of a given size if such an effect truly exists, but also pay attention to legal requirements and ethical and practical considerations to keep the number of animals as small as possible. The required sample size should be determined before the start of the study with a formal sample size calculation, of which the fundamental elements of statistical significance (α), effect size (δ), power (1–β), and standard deviation of the measurements have been explained in numerous articles [13],[21]. Unfortunately, the assumptions on variation of the measurements are often based on incomplete data, and small errors can lead to a study that is either under- or overpowered. From an ethical point of view, underpowered studies are undesirable, as they might lead to the false conclusion that the intervention is without efficacy, and all included animals will have been used to no benefit. Overpowered studies would also be unethical, but these are much less prevalent.

### Monitoring of Physiological Parameters

Depending on the disease under investigation, a range of physiological variables may affect outcome, and inadequate control of these factors may lead to erroneous conclusions. Whether or not physiological parameters should be assessed, and for how long, therefore depends on the model and on the tested condition.

### Eligibility Criteria and Drop-Outs

Because of their complexity, many animal models are inherently vulnerable to complications—such as inadvertent blood loss during surgery to induce cerebral or myocardial ischemia—that are not related to the treatment under study but that may have a large effect on outcome. Given the explanatory character of preclinical studies, it is justifiable to exclude animals with such complications from the analyses of treatment effects, provided that the eligibility criteria are predefined and not determined on a post-hoc basis, and that the person responsible for the exclusion of animals is unaware of the treatment assignment.

In clinical trials, inclusion and exclusion criteria are usually applied before enrolment in the study, but for the reason above, in animal studies it is justifiable also to apply these criteria during the course of the study. However, these should be limited to complications that are demonstrably not related to the intervention under study, as this may otherwise lead to attrition bias. For example, if a potential novel treatment for colorectal cancer increases instead of reduces tumour progression, thereby weakening the animals and increasing their susceptibility to infections, exclusion of animals dying prematurely because of respiratory tract infections may lead to selective exclusion of animals with the largest tumours and mask the detrimental effect of the novel intervention.

### Statistical Analysis

The statistical analysis of the results of animal experiments has been given elaborate attention in review articles and books [22]. However, even when data appear simple and their analysis straightforward, inadequate techniques are often used. Common examples include the use of a *t*-test for nonparametric data, calculating means and standard deviations for ordinal data, and treating multiple observations from one animal as independent.

In clinical trials, an intention-to-treat analysis is generally favoured because it avoids bias associated with nonrandom loss of participants [13]. As explained above, the explanatory character of most studies justifies the use of an analysis restricted to data from animals that have fulfilled all eligibility criteria, provided that all animals excluded from the analysis are accounted for and that those exclusions have been made without knowledge of treatment group allocation.

### Control of Study Conduct

The careers of investigators at academic institutions and in industry depend in part on the number and impact of their publications, and these investigators may be all too aware of the fact that the prospect of their work being published increases when positive results are obtained. This underscores not only the importance of randomisation, allocation concealment, and blinding, but also the need for adequate monitoring and auditing of laboratory experiments by third parties. Indeed, adopting a multicentre approach to animal studies has been proposed, as a way of securing transparent quality control [23].

### Bias in Animal Studies

The presence of bias in animal studies has been tested most extensively in studies of acute ischemic stroke, probably because in this field the gap between the laboratory and the clinic is both very large and well recognised [8]. In systematic reviews of different interventions tested in animal models of acute ischemic stroke, other emergencies, Parkinson's disease, multiple sclerosis, or amyotrophic lateral sclerosis, generally about a third or less of the studies reported random allocation to the treatment group, and even fewer studies reported concealment of treatment allocation or blinded outcome assessment [2],[16],[19],[24],[25]. Even when reported, the methods used for randomisation and blinding were rarely given. A priori sample size calculations were reported in 0%–3% of the studies (Table 2).

**Table 2:** Randomisation, blinded outcome assessment, and sample size calculation in systematic reviews of animal studies.

Disease Modeled	Year of Publication	Number of Publications	Randomisation, *n* (%)	Blinded Outcome Assessment, *n* (%)	A Priori Sample Size Calculation, *n* (%)
Heart failure [24]	2003	9	6 (67)	9 (100)	0 (0)
Emergency medicine [33]	2003	290	94 (32)	31 (11)	N/A
Ischemic stroke [19]	2005	45	19 (42)	18 (40)	0 (0)
Ischemic stroke [49]	2005	73	17 (23)	9 (12)	N/A
Ischemic stroke [50]	2005	25	8 (32)	1 (4)	N/A
Ischemic stroke [51]	2006	27	2 (7)	1 (4)	N/A
Traumatic brain injury [2]	2007	17	2 (12)	3 (18)	N/A
Hemorrhage in surgery [2]	2007	8	3 (38)	4 (50)	N/A
Neonatal RDS [2]	2007	56	14 (25)	3 (5)	N/A
Osteoporosis [2]	2007	16	5 (31)	0 (0)	N/A
Ischemic stroke [16] a	2007	288	103 (36)	84 (29)	8 (3)
Parkinson's disease [16]	2007	118	14 (12)	18 (15)	0 (0)
Multiple sclerosis [16]	2007	183	4 (2)	20 (11)	0 (0)
ALS [45]	2007	85	21 (25)	21 (25)	1 (1)
Brain injury [52]	2008	18	12 (67)	7 (39)	N/A
Ischemic stroke [25]	2008	9	3 (33)	4 (44)	2 (22)
Ischemic stroke [53]	2009	19	1 (5)	5 (26)	0 (0)

a Summarises the data of six systematic reviews of treatment strategies for acute ischemic stroke. There is an overlap of 18 publications between references [16] and [19].

ALS, amyotrophic lateral sclerosis; N/A, data not available; RDS, respiratory distress syndrome.

Complications of the disease and/or treatment under study were reported in 19% of the studies of hypothermia for acute ischemic stroke. All but one of these complications concerned premature death, and about 90% of these animals were excluded from the analyses [20]. In another review of several treatment strategies for acute ischemic stroke, only one of 45 studies mentioned predefined inclusion and exclusion criteria, and in just 12 articles (27%) exclusion of animals from analysis was mentioned and substantiated. It is difficult to believe that in every other study every single experiment went as smoothly as the investigators had planned [19].

Two factors limit the interpretation of the above-mentioned data. First, the assessment of possible confounders in systematic reviews was based on what was reported in the articles, and may have been incomplete because the authors considered these aspects of study design not sufficiently relevant to be mentioned. In addition, definitions of randomisation, allocation concealment, and blinding might vary across studies, and, for example, randomly picking animals from their cages may have been called “randomisation.” Indeed, a survey of a sample of authors of publications included in such reviews suggested that this was sometimes the case [26].

### Quality Checklists

At least four different but largely overlapping study-quality checklists have been proposed for use in animal studies of focal cerebral ischemia. These checklists have included items relating first to the range of circumstances under which efficacy has been shown and second to the characteristics that might act as a source of bias in individual experiments [16].

Assessment of overall methodological quality of individual studies with these checklists is limited by controversy about the composition of the checklists and, more importantly, because the weight of each of the individual components has remained uncertain. For example, in the most frequently used CAMARADES checklist, “adequate allocation concealment” may have a much larger impact on effect size than “compliance with regulatory requirements” [16].

### Does Methodological Quality Matter?

Several systematic reviews and meta-analyses have provided empirical evidence that inadequate methodological approaches in controlled clinical trials are associated with bias. Clinical trials in which authors did not report randomisation, adequately conceal treatment allocation, or use double blinding yielded larger estimates of treatment effects than trials in which these study quality issues were reported [12],[27]–[32].

The impact of methodological quality on the effect size in animal studies has been examined less extensively. In animal studies testing interventions in emergency medicine, the odds of a positive result were more than three times as large if the publication did not report randomisation or blinding as compared with publications that did report these methods [33]. In systematic reviews of FK-506 or hypothermia for acute ischemic stroke, an inverse relation was found between effect size and study quality, as assessed by a ten-item study-quality checklist [20],[34]. The same review on hypothermia found large overstatements of the reduction in infarct volume in animal stroke studies without randomisation or blinded outcome assessment when they were compared with randomised or blinded studies, but a meta-analysis of 13 meta-analyses in experimental stroke describing outcomes in a total of 15,635 animals found no statistically significant effect of these quality items on effect size. In this meta-meta-analysis, only allocation concealment was associated with a larger effect size [35].

A limitation of the meta-analyses assessing the effect of study quality aspects on effect size is the fact that no consideration has been given to possible interactions between quality items, and that only univariate analyses were performed. However, individual quality aspects that may affect the results of meta-analyses of animal studies are unlikely to operate independently. For example, nonrandomised studies may be more likely than randomised studies to disregard other quality issues, such as allocation concealment or blinding, or to use shorter delays for the initiation of treatment, all of which may affect study results. The relative importance of the various possible sources of bias is therefore not yet known and is the subject of ongoing research.

## External Validity

Even if the design and conduct of an animal study are sound and eliminate the possibility of bias, the translation of its results to the clinic may fail because of disparities between the model and the clinical trials testing the treatment strategy. Common causes of such reduced external validity are listed in Box 2 and are not limited to differences between animals and humans in the pathophysiology of disease, but also include differences in comorbidities, the use of co-medication, timing of the administration and dosing of the study treatment, and the selection of outcome measures. Whereas the issues for internal validity probably apply to the majority of animal models regardless of the disease under study, the external validity of a model will largely be determined by disease-specific factors.

**Box 2. Common Causes of Reduced External Validity of Animal Studies**

- The induction of the disease under study in animals that are young and otherwise healthy, whereas in patients the disease mainly occurs in elderly people with co-morbidities.
- Assessment of the effect of a treatment in a homogeneous group of animals versus a heterogeneous group of patients.
- The use of either male or female animals only, whereas the disease occurs in male and female patients alike.
- The use of models for inducing a disease or injury with insufficient similarity to the human condition.
- Delays to start of treatment that are unrealistic in the clinic; the use of doses that are toxic or not tolerated by patients.
- Differences in outcome measures and the timing of outcome assessment between animal studies and clinical trials.

### Stroke Models

As mentioned above, the translation of efficacy from animal studies to human disease has perhaps been least successful for neurological diseases in general and for ischaemic stroke in particular. As there is also no other animal model of disease that has been more rigorously subjected to systematic review and meta-analysis, stroke serves as a good example of where difficulties in translation might arise.

The incidence of stroke increases with age, and stroke patients commonly have other health problems that might increase their stroke risk, complicate their clinical course, and affect functional outcome. Of patients with acute stroke, up to 75% and 68% have hypertension and hyperglycaemia, respectively [9],[36]. While it is important to know whether candidate stroke drugs retain efficacy in the face of these comorbidities, only about 10% of focal ischaemia studies have used animals with hypertension, and fewer than 1% have used animals with induced diabetes. In addition, animals used in stroke models were almost invariably young, and female animals were highly underrepresented. Over 95% of the studies were performed in rats and mice, and animals that are perhaps biologically closer to humans are hardly ever used [16],[19]. Moreover, most animal studies have failed to acknowledge the inevitable delay between the onset of symptoms and the possibility to start treatment in patients. In a systematic review of animal studies of five different neuroprotective agents that had also been tested in 21 clinical trials including a total of more than 12,000 patients with acute ischaemic stroke, the median time between the onset of ischaemia and start of treatment in the animal studies was just 10 minutes, which is infeasible in the clinic [19]. In the large majority of clinical trials, functional outcome is the primary measure of efficacy, whereas animal studies usually rely on infarct volume. Several studies have suggested that in patients the relation between infarct volume and functional outcome is moderate at best [37],[38]. Finally, the usual time of outcome assessment of 1–3 days in animal models contrasts sharply with that of 3 months in patients [19]. For these reasons, it is not surprising that, except for thrombolysis, all treatment strategies proven effective in the laboratory have failed in the clinic.

### Other Acute Disease Models

Differences between animal models and clinical trials similar to those mentioned above have been proposed as causes of the recurrent failure of a range of strategies to reduce lethal reperfusion injury in patients with acute myocardial infarction [6],[7]. The failure to acknowledge the presence of often severe comorbidities in patients, and short and clinically unattainable onset-to-treatment delays, have also limited the external validity of animal models of traumatic brain injury [2].

### Chronic Disease Models

The external validity of models of chronic and progressive diseases may also be challenged by other factors. For the treatment of Parkinson's disease, researchers have mainly relied on injury-induced models that mimic nigrostriatal dopamine deficiency but do not recapitulate the slow, progressive, and degenerative nature of the disease in humans. Whereas in clinical trials interventions were administered over a prolonged period of time in the context of this slowly progressive disease, putative neuroprotective agents were administered before or at the same time as an acute Parkinson's disease-like lesion was induced in the typical underlying animal studies [39].

Based on the identification of single point-mutations in the gene encoding superoxide dismutase 1 (SOD1) in about 3% of the patients with amyotrophic lateral sclerosis (ALS), mice carrying 23 copies of the human SOD1G93A transgene are considered the standard model for therapeutic studies of ALS. Apart from the fact that this model may be valid only for patients with SOD1 mutations, the mice may suffer from a phenotype that is so aggressive and so overdriven by its 23 copies of the transgene that no pharmacological intervention outside of the direct inhibition of SOD1 will ever affect ALS-related survival. In addition, it has been suggested that these mice may be more susceptible to infections and other non-ALS related illnesses and that it is this illness rather than the ALS that is alleviated by the experimental treatment. Consistent with this hypothesis, several of the compounds reported as efficacious in SOD1G93A mice are broad-spectrum antibiotics and general anti-inflammatory agents [40].

## Publication Bias

Decisions to assess the effect of novel treatment strategies in clinical trials are, ideally, based on an understanding of all publicly reported information from preclinical studies. Systematic review and meta-analysis are techniques developed for the analysis of data from clinical trials and may be helpful in the selection of the most promising strategies [16]. However, if studies are published selectively on the basis of their results, even a meta-analysis based on a rigorous systematic review will be misleading.

The presence of bias in the reporting of clinical trials has been studied extensively. There is strong empirical evidence that clinical studies reporting positive or significant results are more likely to be published, and that outcomes that are statistically significant have higher odds of being reported in full rather than as an abstract. Such publication bias will lead to overestimation of treatment effects and can make the readily available evidence unreliable for decision making [41].

Unfortunately, the presence of publication bias in animal studies has received much less attention. In a recent systematic review of studies testing the efficacy of interventions in animal models of human disease, only six reported testing for the presence of publication bias, and such bias was found in four [34],[42]–[46]. No study gave quantitative estimates of the impact on effect size of publication bias [47].

In a subsequent meta-analysis of 525 publications [47] included in systematic reviews of 16 interventions tested in animal studies of acute ischaemic stroke, Egger regression and Trim and Fill analysis suggested that publication bias was widely prevalent. The analyses suggested that publication bias might account for around one-third of the efficacy reported in systematic reviews of animal stroke studies. Because this meta-analysis included all reported experiments testing an effect of an intervention on infarct size, and not just the experiment with the largest effect size from each publication, at least some experiments testing ineffective doses (e.g., at the lower end of a dose-response curve) were included. For this reason, this meta-analysis is more likely to underestimate than to overestimate the effect of publication bias. It is therefore probably more revealing that of the 525 publications, only ten (2%) did not report at least one significant effect on either infarct volume or neurobehavioural score [47]. Although unproven, it appears unlikely that the animal stroke literature is uniquely susceptible to publication bias.

Nonpublication of the results of animal studies is unethical not only because it deprives researchers of the accurate data they need to estimate the potential of novel therapies in clinical trials, but also because the included animals are wasted because they do not contribute to accumulating knowledge. In addition, research syntheses that overstate biological effects may lead to further unnecessary animal experiments testing poorly founded hypotheses.

## Practical Improvement Strategies

Although there is no direct evidence of a causal relationship, it is likely that the recurrent failure of apparently promising interventions to improve outcome in clinical trials has in part been caused by inadequate internal and external validity of preclinical studies and publication bias favouring positive studies. On the basis of ample empirical evidence from clinical trials and some evidence from preclinical studies, we suggest that the testing of treatment strategies in animal models of disease and its reporting should adopt standards similar to those in the clinic to ensure that decision making is based on high-quality and unbiased data. Aspects of study quality that should be reported in any manuscript are listed in Box 3.

**Box 3. Aspects of Study Quality to Be Reported in the Manuscript**

- **Sample size calculation:** How the sample size was determined, and which assumptions were made.
- **Eligibility criteria:** Inclusion and exclusion criteria for enrolment.
- **Treatment allocation:** The method by which animals were allocated to experimental groups. If this allocation was by randomisation, the method of randomisation.
- **Allocation concealment:** The method to implement the allocation sequence, and if this sequence was concealed until assignment.
- **Blinding:** Whether the investigators and other persons involved were blinded to the treatment allocation, and at which points in time during the study.
- **Flow of animals:** Flow of animals through each stage of the study, with a specific attention to animals excluded from the analyses. Reasons for exclusion from the analyses.
- **Control of physiological variables:** Whether and which physiological parameters were monitored and controlled.
- **Control of study conduct:** Whether a third party controlled which parts of the conduct of the study.
- **Statistical methods:** Which statistical methods were used for which analysis.

Recommendations based on [13],[17].

Not only should the disease or injury itself reflect the condition in humans as much as possible, but age, sex, and comorbidities should also be modelled where possible. The investigators should justify their selection of the model and outcome measures. In turn, human clinical trials should be designed to replicate, as far as is possible, the circumstances under which efficacy has been observed in animals. For an adequate interpretation of the potential and limitations of a novel treatment strategy, a systematic review and meta-analysis of all available evidence from preclinical studies should be performed before clinical trials are started. Evidence of benefit from a single laboratory or obtained in a single model or species is probably not sufficient.

Finally, the recognition of substantial publication bias in the clinical literature has led to the introduction of clinical trial registration systems to ensure that those summarising research findings are at least aware of all relevant clinical trials that have been performed [48]. Given that a framework regulating animal experimentation already exists in many countries, we suggest that this might be exploited to allow the maintenance of a central register of experiments performed, and registration referenced in publications.

**Five Key Papers in the Field**

**Hackam 2006** [1]: Shows that about a third of highly cited animal research translates at the level of human randomised trials.

**Sena 2007** [16]: Proposes minimum standards for the range and quality of pre-clinical animal data before these are taken to clinical trials.

**Dirksen 2007** [6]: Provides an overview of the various strategies that inhibit reperfusion injury after myocardial infarction and discusses potential mechanisms that may have contributed to the discrepancy between promising pre-clinical data and the disappointing results in randomised clinical trials.

**Scott 2008** [40]: Elaborate study suggesting that the majority of published effects of treatments for amyotrophic lateral sclerosis are most likely measurements of noise in the distribution of survival means as opposed to actual drug effect.

**Sena 2010** [47]: The first study to estimate the impact of publication bias on the efficacy reported in systematic reviews of animal studies.

## Abbreviations

ALS: amyotrophic lateral sclerosis
CAMARADES: Collaborative Approach to Meta-Analysis And Review of Animal Data from Experimental Stroke
CONSORT: CONsolidated Standards Of Reporting Trials
